# Supplementary material for: The cytochrome c oxidase subunit COX6B1 is required for redox-sensitive early assembly and late stabilization of complex IV
Source: J Biol Chem. 2025 Dec 17;302(2):111070. doi: 10.1016/j.jbc.2025.111070 (PMC12870770; doi:10.1016/j.jbc.2025.111070)
Supplement: Supplementary Material 1 [file mmc1.pdf]

Supporting information 1

The cytochrome c oxidase subunit COX6B1 is required for redox-sensitive early assembly and late stabilization of complex IV

Kristýna Čunátová[1,2,3], Marek Vrbacký[1], Michal Knězů[1], Alena Pecinová[1], Lukáš Alán[1,4], Josef Houštěk[1], Erika Fernández-Vizarra[2,3,5], Tomáš Mráček[1] and Petr Pecina[1*]

[1] Laboratory of Bioenergetics, Institute of Physiology, Czech Academy of Sciences, Prague, 14200, Czech Republic
[2] Department of Biomedical Sciences, University of Padova, Padova, 35131, Italy
[3] Veneto Institute of Molecular Medicine, Padova, 35129, Italy
[4] Department of Biology, University of Padova, Padova, 35131, Italy
[5] Department of Biochemistry and Molecular and Cellular Biology, University of Zaragoza, Huesca, 22002, Spain
** Corresponding author*

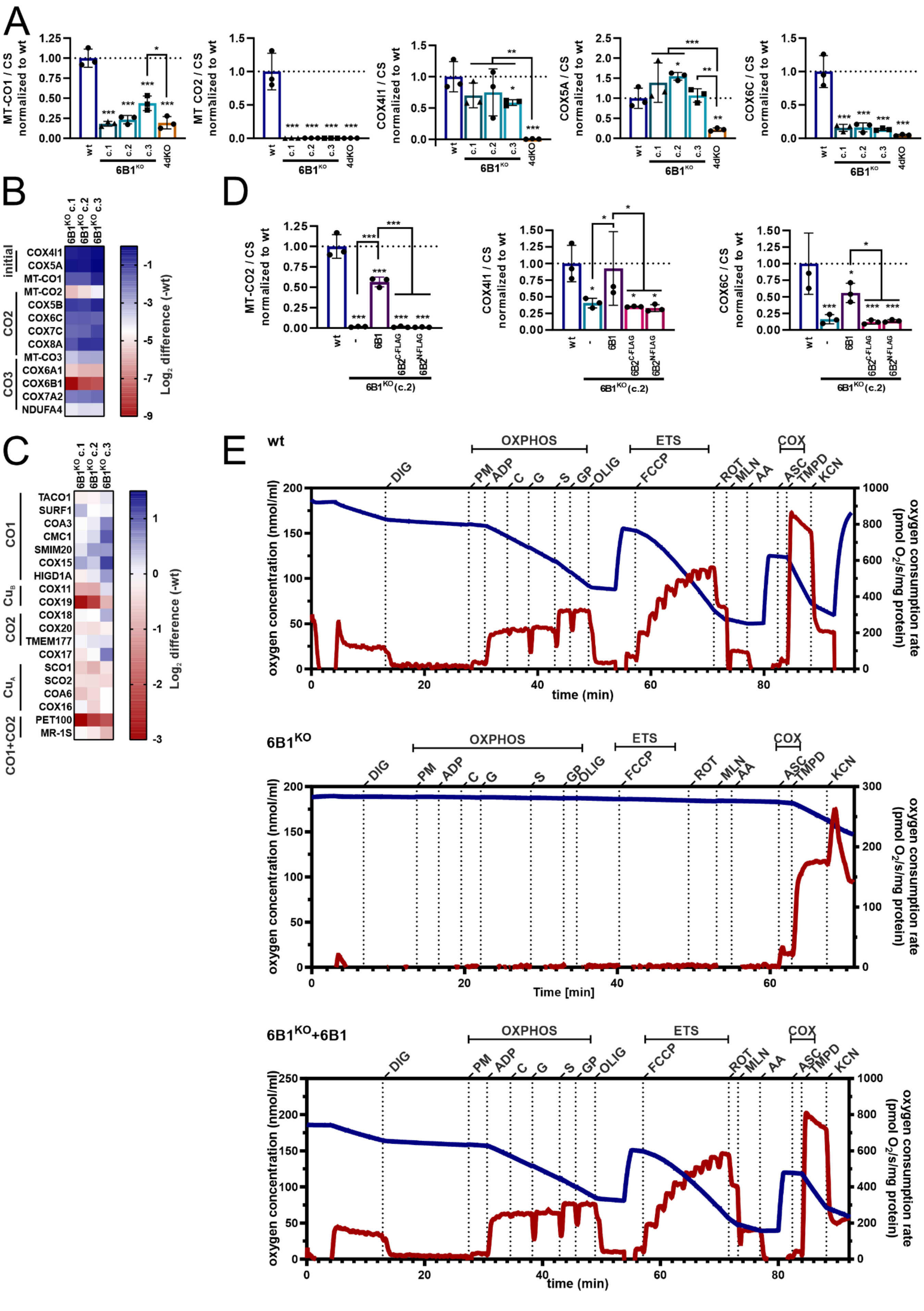
A
MT-CO1 / CS normalized to wt
MT CO2 / CS normalized to wt
COX4I1 / CS normalized to wt
COX5A / CS normalized to wt
COX6C / CS normalized to wt
wt
c.1
c.2
c.3
4dKO
6B1KO
B
6B1KO c.1
6B1KO c.2
6B1KO c.3
initial
COX4I1
COX5A
MT-CO1
CO2
MT-CO2
COX5B
COX6C
COX7C
COX8A
CO3
MT-CO3
COX6A1
COX6B1
COX7A2
NDUFA4
Log2 difference (-wt)
C
CO1
TACO1
SURF1
COA3
CMC1
SMIM20
COX15
HIGD1A
CuB
COX11
COX19
CO2
COX18
COX20
TMEM177
COX17
CuA
SCO1
SCO2
COA6
COX16
CO1+CO2
PET100
MR-1S
D
MT-CO2 / CS normalized to wt
COX4I1 / CS normalized to wt
COX6C / CS normalized to wt
6B1
6B2C-FLAG
6B2N-FLAG
6B1KO(c.2)
E
wt
6B1KO
6B1KO+6B1
OXPHOS
ETS
COX
DIG
PM
ADP
C
G
S
GP
OLIG
FCCP
ROT
MLN
AA
ASC
TMPD
KCN
oxygen concentration (nmol/ml)
oxygen consumption rate (pmol O2/s/mg protein)
time (min)
Time [min]

Figure S1: COX6B1 knock-out blocks early human cIV assembly

(A) Quantification of the MT-CO1, MT-CO2, COX4I1, COX5A, COX6C signals from SDS-PAGE/WB analysis normalized to CS (representative images are shown in Fig1B). One-way ANOVA (* $p < 0.05$; ** $p < 0.01$; *** $p < 0.001$) was performed ($n = 3$, mean ± SD).

(B) Differential content of cIV subunits between wt and individual $6B1^{KO}$ clones. Heatmap represents LFQ-MS analysis (wt: $n = 4$; $6B1^{KO}$ c.1, c.2 and c.3: $n = 2$ per each) of analyzed subunits of cIV modules. COX6B1 protein missing in $6B1^{KO}$ was visualized thanks to the imputation step performed during the Perseus analysis of the LFQ-MS data.

(C) Differential content of cIV assembly factors (AFs) between wt and individual $6B1^{KO}$ clones. Heatmap represents LFQ-MS analysis (wt: $n = 4$; $6B1^{KO}$ c.1, c.2 and c.3: $n = 2$ per each) of analyzed cIV assembly factors.

(D) Quantification of the MT-CO2, COX4I1 and COX6C signals from SDS-PAGE/WB analysis normalized to CS (representative images are shown in Fig1F). One-way ANOVA (* $p < 0.05$; ** $p < 0.01$; *** $p < 0.001$) was performed ($n = 3$, mean ± SD).

(E) Representative trace of respirometric measurement of wt, $6B1^{KO}$ and $6B1^{KO}$+6B1 cells (relevant for Fig1G). Experimental trace recorded by Oxygraph-2k (Oroboros) shows the actual O2 concentration (blue, left Y axis) and rate of oxygen consumption (red, right Y axis). Additions of substrates and inhibitors are marked by vertical dashed lines and abbreviations above the trace (digitonin - DIG, pyruvate + malate - PM, ADP - ADP, cytochrome c - C, glutamate - G, succinate - S, glycerol-3 phosphate - GP, oligomycin - OLIG, FCCP - FCCP, rotenone - ROT, malonate - MLN, antimycin A - AA, ascorbate – A, TMPD - T, and KCN - KCN).

Related to Fig1

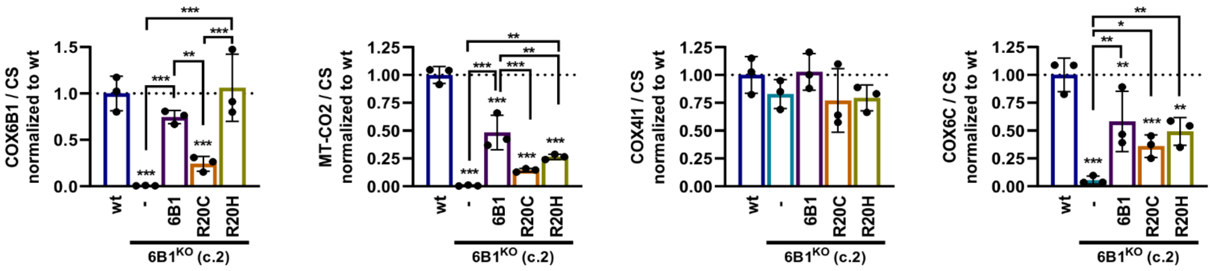

Figure S2: R20C pathogenic variant of COX6B1 protein is less stable than R20H
Quantification of the COX6B1, MT-CO2, COX4I1, COX6C signals from SDS-PAGE/WB analysis normalized to CS (representative images are shown in Fig2C). One-way ANOVA (* $p < 0.05$; ** $p < 0.01$; *** $p < 0.001$) was performed ($n = 3$, mean ± SD).
Related to Fig2

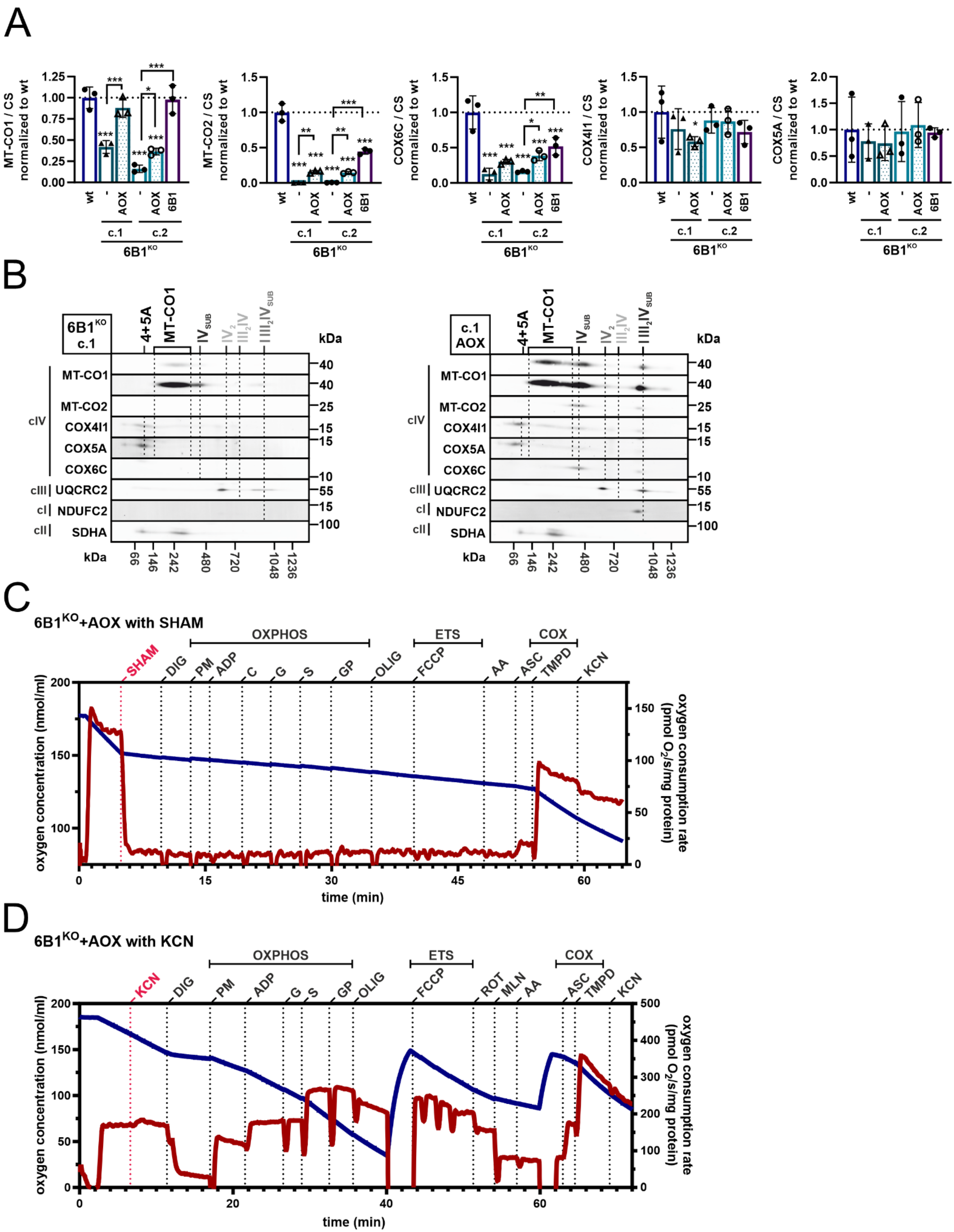

A
MT-CO1 / CS normalized to wt
MT-CO2 / CS normalized to wt
COX6C / CS normalized to wt
COX4I1 / CS normalized to wt
COX5A / CS normalized to wt
wt
AOX
6B1
c.1
c.2
6B1KO
B
6B1KO c.1
c.1 AOX
4+5A
MT-CO1
IVSUB
IV2
III2IV
III2IVSUB
kDa
MT-CO1
MT-CO2
COX4I1
COX5A
COX6C
UQCRC2
NDUFC2
SDHA
cIV
cIII
cI
cII
66
146
242
480
720
1048
1236
C
6B1KO+AOX with SHAM
OXPHOS
ETS
COX
SHAM
DIG
PM
ADP
C
G
S
GP
OLIG
FCCP
AA
ASC
TMPD
KCN
oxygen concentration (nmol/ml)
oxygen consumption rate (pmol O2/s/mg protein)
time (min)
D
6B1KO+AOX with KCN
OXPHOS
ETS
COX
KCN
DIG
PM
ADP
G
S
GP
OLIG
FCCP
ROT
MLN
AA
ASC
TMPD
KCN
oxygen concentration (nmol/ml)
oxygen consumption rate (pmol O2/s/mg protein)
time (min)

Figure S3: Alternative oxidase expression ameliorates cIV composition and function in COX6B1 deficient cells

(A) Quantification of the MT-CO1, MT-CO2, COX4I1, COX5A, COX6C signals from SDS-PAGE/WB analysis normalized to CS (representative images are shown in Fig4A). One-way ANOVA (* $p < 0.05$; ** $p < 0.01$; *** $p < 0.001$) was performed ($n = 3$, mean ± SD).

(B) 2D (BN/SDS)-PAGE/WB detection of cIV (MT-CO1, MT-CO2, COX4I1, COX5A, and COX6C antibodies), cIII (UQCRC2 antibody), and cI (NDUFC2 antibody) in $6B1^{KO}$ c.1 (left) and $6B1^{KO}$ c.1 +AOX (right) mitochondrial fraction. Antibody against cII (SDHA) was used as a loading control.

(C) Representative trace of respirometric measurement of cIV-specific respiration of $6B1^{KO}$+AOX (relevant for Fig4E, Fig4F). Experimental trace recorded by Oxygraph-2k (Oroboros) shows the actual O2 concentration (blue, left Y axis) and rate of oxygen consumption (red, right Y axis). Additions of substrates and inhibitors are marked by vertical dashed lines and abbreviations above the trace (SHAM – salicylhydroxamic acid/inhibitor of AOX, digitonin - DIG, pyruvate + malate - PM, ADP - ADP, cytochrome c - C, glutamate - G, succinate - S, glycerol-3 phosphate - GP, oligomycin - OLIG, FCCP - FCCP, antimycin A - AA, ascorbate – A, TMPD - T, and KCN - KCN).

(D) Representative trace of respirometric measurement of AOX-specific respiration of $6B1^{KO}$+AOX. Experimental trace recorded by Oxygraph-2k (Oroboros) shows the actual O2 concentration (blue, left Y axis) and rate of oxygen consumption (red, right Y axis). Additions of substrates and inhibitors are marked by vertical dashed lines and abbreviations above the trace (KCN – KCN, digitonin - DIG, pyruvate + malate - PM, ADP - ADP, cytochrome c - C, glutamate - G, succinate - S, glycerol-3 phosphate - GP, oligomycin - OLIG, FCCP - FCCP, antimycin A - AA, ascorbate – A, TMPD - T, and KCN - KCN).

Related to Fig4

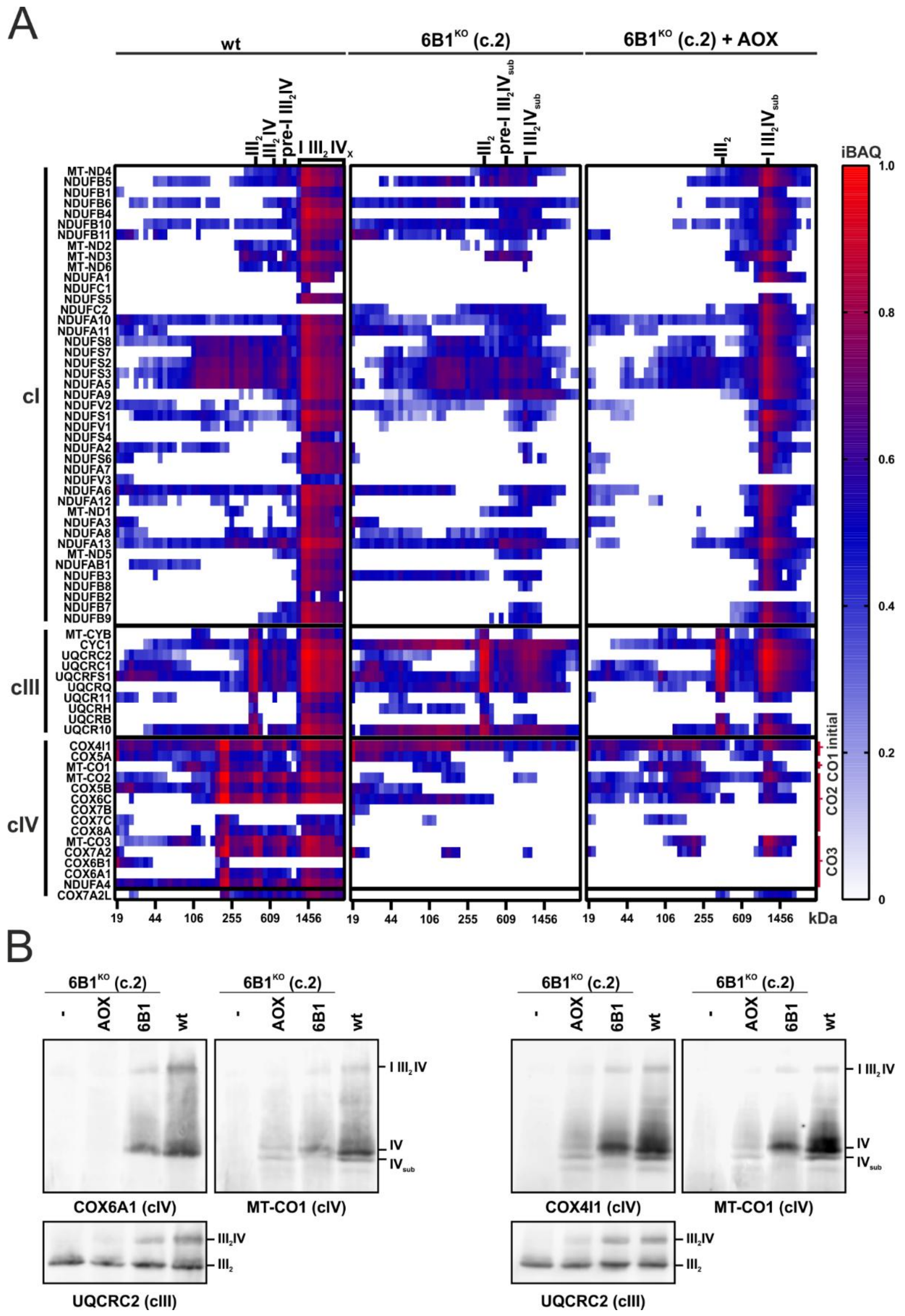

Figure S4: Alternative oxidase expression restores cIV assembly in COX6B1 deficient cells
(A) Complexome profiling analysis of cI, cIII and cIV subunits in wt, $6B1^{KO}$, and $6B1^{KO}$+AOX respectively (relevant for Fig5B). Heat-map representation of relative iBAQ values.
(B) Blue-native (BN)-PAGE/WB detection of cIV using COX6A1, MT-CO1 and COX4 antibodies. Antibody against cIII (UQCRC2) was used as a loading control.
Related to Fig5
