# Supplementary material for: The cytochrome c oxidase subunit COX6B1 is required for redox-sensitive early assembly and late stabilization of complex IV
Source: J Biol Chem. 2025 Dec 17;302(2):111070. doi: 10.1016/j.jbc.2025.111070 (PMC12870770; doi:10.1016/j.jbc.2025.111070)
Supplement: Supplementary Material 2 [file mmc2.pdf]

Supporting information 2 – Validation information on antibodies

Primary Antibody	Source	Identifier	Validation
NDUFA9 antibody	Abcam	Cat# ab14713, RRID:AB_301431	Knock-out validated and literature consistency*
NDUFC2 antibody	Proteintech	Cat# 15573-1-AP, RRID:AB_10666733	Literature consistency*
SDHA antibody	Abcam	Cat# ab14715, RRID:AB_301433	Knock-out validated and literature consistency*. Also, KO validated in our previous study (PMID: 39184436)
UQCRC2 antibody	Abcam	Cat# ab14745, RRID:AB_2213640	No genetic validation but overall literature consistency of the product*
UQCRC2 antibody	Proteintech	Cat# 14742-1-AP, RRID:AB_2241442	Knock-down validated*
MTCO1 antibody	Abcam	Cat# ab14705, RRID:AB_2084810	Literature consistency*
MTCO2 (Cytochrome C oxidase subunit II) antibody	Abcam	Cat# ab110258, RRID:AB_10887758	Literature consistency*
COX4I1 (Anti-COX IV) antibody	Abcam	Cat# ab14744, RRID:AB_301443	KO validated in our previous studies (PMID: 33578848, 32075102) and literature consistency*
COX5A antibody	Abcam	Cat# ab110262, RRID: AB_10861723	Literature consistency*
COX6A1 antibody	Proteintech	Cat# 11460-1-AP, RRID: AB_2085445	Knock-down/knock-out validated in our previous study (PMID: 39184436) and present manuscript, and literature consistency*.
COX6B1 (Cytochrome C Oxidase subunit VIb) antibody	Proteintech	Cat# 11425-1-AP, RRID: AB_2085449	Knock-out validated and literature consistency*. Also, KO validated
COX6C (Cytochrome C Oxidase subunit VIc) antibody	Abcam	Cat# ab110267, RRID:AB_10861117	Literature consistency*
ATP5F1B (ATPB) antibody	Abcam	Cat# ab14730, RRID:AB_301438	Knock-out validated in our previous study (PMID: 39184436), literature consistency*

FLAG antibody	Sigma-Aldrich	Cat# F1804, RRID:AB_262044	Literature consistency*
HA Tag antibody	Thermo Fisher Scientific	Cat# 26183, RRID:AB_10978021	Literature consistency*
CS (Citrate synthetase) antibody	Abcam	Cat# ab129095, RRID:AB_11143209	No genetic validation but overall literature consistency of the product

* see manufacturer information
