# Supplementary material for: The cytochrome c oxidase subunit COX6B1 is required for redox-sensitive early assembly and late stabilization of complex IV
Source: J Biol Chem. 2025 Dec 17;302(2):111070. doi: 10.1016/j.jbc.2025.111070 (PMC12870770; doi:10.1016/j.jbc.2025.111070)
Supplement: Key resources table [file mmc3.docx]

# Key resources table

| REAGENT or RESOURCE | SOURCE | IDENTIFIER |
| --- | --- | --- |
| Chemicals, peptides, and recombinant proteins | | |
| Metafectene Pro | Biontex | Cat# T040-2.0 |
| Benzonase® Nuclease | Merck | Cat# 70664 |
| Immobilon®-FL PVDF membrane | Merck | Cat# 05317 |
| NativePAGE Cathode Buffer Additive (20X) | Thermo Fisher Scientific | Cat#BN2002 |
| NativePAGE Running Buffer (20X) | Thermo Fisher Scientific | Cat#BN2001 |
| NativePAGE 3-12% Bis-Tris Gels | Thermo Fisher Scientific | Cat# BN1001BOX |
| Complex IV immunocapture kit | Abcam | Cat# ab109801 |
| Deposited data | | |
| Label-free quantification mass spectrometry data | Proteomics Identifications (PRIDE) | PXD061621 |
| Complexome profiling mass spectrometry data | Proteomics Identifications (PRIDE) | PXD061618 |
| Experimental models: Cell lines | | |
| Wild-type HEK293 cell line (wt) | ATCC | Cat#: CRL-1573, RRID:CVCL_0045 |
| *COX4I1/COX4I2* knock-out (KO) HEK293 cell line (4dKO) | Čunátová et al. 2021 | N/A |
| *COX6B1* KO HEK293 cell line (6B1^KO^ c.1) | Čunátová et al. 2024 | N/A |
| *COX6B1* KO HEK293 cell line (6B1^KO^ c.2) | Čunátová et al. 2024 | N/A |
| *COX6B1* KO HEK293 cell line (6B1^KO^ c.3) | Čunátová et al. 2024 | N/A |
| 6B1^KO^ c.1 with stable AOX xeno-expression (6B1^KO^+AOX) | this paper | N/A |
| 6B1^KO^ c.2 with stable AOX xeno-expression (6B1^KO^+AOX) | Čunátová et al. 2024 | N/A |
| 6B1^KO^ c.2 with stable COX6B1-R20C-C terminal FLAG expression (6B1^KO^+R20C) | this paper | N/A |
| 6B1^KO^ c.2 with stable COX6B1-R20H-C terminal FLAG expression (6B1^KO^+R20H) | this paper | N/A |
| 6B1^KO^ c.2 with stable COX6B1-C terminal FLAG expression (6B1^KO^+6B1) | this paper | N/A |
| 6B1^KO^ c.2 with stable COX6B2-C terminal FLAG expression (6B1^KO^+6B2) | this paper | N/A |
| 6B1^KO^ c.2 with stable COX6B2-N terminal FLAG expression (6B1^KO^+6B2) | this paper | N/A |
| Oligonucleotides | | |
| Site-directed mutagenesis primer for COX6B1-R20C:  5'-ctggttggggaagcagctgtcaaaagggg-3', 5'-ccccttttgacagctgcttccccaaccag-3' | this paper | N/A |
| Site-directed mutagenesis primer for COX6B1-R20H:  5'-ttctggttggggaagtggctgtcaaaagggg-3', 5'-ccccttttgacagccacttccccaaccagaa-3' | this paper | N/A |
| Recombinant DNA | | |
| pcDNA™3.1 (+) Mammalian Expression Vector | Thermo Fisher Scientific | Cat# V79020 |
| pcDNA™3.1 (+) Mammalian Expression Vector for AOX expression | Čunátová et al. 2024 | N/A |
| pcDNA™3.1 (+) Mammalian Expression Vector for COX6B1-C terminal FLAG expression | this paper | N/A |
| pcDNA™3.1 (+) Mammalian Expression Vector for COX6B2-C terminal FLAG expression | this paper | N/A |
| pcDNA™3.1 (+) Mammalian Expression Vector for COX6B2-N terminal FLAG expression | this paper | N/A |
| pcDNA™3.1 (+) Mammalian Expression Vector for COX6B1-R20C-C terminal FLAG expression | this paper | N/A |
| pcDNA™3.1 (+) Mammalian Expression Vector for COX6B1-R20H-C terminal FLAG expression | this paper | N/A |
| Software and algorithms | | |
| MaxQuant (v. 1.5.3.28) - MaxLFQ algorithm | Tyanova et al. 2016a, Cox et al. 2014 | RRID:SCR_014485 |
| Perseus (v. 2.0.11.0.) | Tyanova et al. 2016b | RRID:SCR_015753 |
| GraphPad Prism 8 software | GraphPad Software | RRID:SCR_002798 |
| Image Lab software | Bio-Rad | RRID:SCR_014210 |
